# 4D MUSIC CMR: value-based imaging of neonates and infants with congenital heart disease

**DOI:** 10.1186/s12968-017-0352-8

**Published:** 2017-04-03

**Authors:** Kim-Lien Nguyen, Fei Han, Ziwu Zhou, Daniel Z. Brunengraber, Ihab Ayad, Daniel S. Levi, Gary M. Satou, Brian L. Reemtsen, Peng Hu, J. Paul Finn

**Affiliations:** 1 Diagnostic Cardiovascular Imaging Laboratory, Department of Radiological Sciences, David Geffen School of Medicine at UCLA, Los Angeles, CA USA; 2 Division of Cardiology, David Geffen School of Medicine at UCLA and VA Greater Los Angeles Healthcare System, Los Angeles, CA USA; 3 Department of Biomedical Physics, University of California, Los Angeles, CA USA; 4 Department of Anesthesiology, David Geffen School of Medicine at UCLA, Los Angeles, CA USA; 5 Division of Pediatric Cardiology, David Geffen School of Medicine at UCLA, Los Angeles, CA USA; 6 Division of Cardiothoracic Surgery, David Geffen School of Medicine at UCLA, Los Angeles, CA USA; 7 Department of Radiological Sciences, University of California at Los Angeles, Peter V. Ueberroth Building Suite 3371, 10945 Le Conte Ave., Los Angeles, CA 90095-7206 USA

**Keywords:** Congenital heart disease, Magnetic resonance angiography, Cardiovascular magnetic resonance, Ferumoxytol, 4-D imaging, Magnetic resonance angiography, Neonates, Infants, Congenital heart disease, Ferumoxytol, 4D

## Abstract

**Background:**

4D Multiphase Steady State Imaging with Contrast (MUSIC) acquires high-resolution volumetric images of the beating heart during uninterrupted ventilation. We aim to evaluate the diagnostic performance and clinical impact of 4D MUSIC in a cohort of neonates and infants with congenital heart disease (CHD).

**Methods:**

Forty consecutive neonates and infants with CHD (age range 2 days to 2 years, weight 1 to 13 kg) underwent 3.0 T CMR with ferumoxytol enhancement (FE) at a single institution. Independently, two readers graded the diagnostic image quality of intra-cardiac structures and related vascular segments on FE-MUSIC and breath held FE-CMRA images using a four-point scale. Correlation of the CMR findings with surgery and other imaging modalities was performed in all patients. Clinical impact was evaluated in consensus with referring surgeons and cardiologists. One point was given for each of five key outcome measures: 1) change in overall management, 2) change in surgical approach, 3) reduction in the need for diagnostic catheterization, 4) improved assessment of risk-to-benefit for planned intervention and discussion with parents, 5) accurate pre-procedural roadmap.

**Results:**

All FE-CMR studies were completed successfully, safely and without adverse events. On a four-point scale, the average FE-MUSIC image quality scores were >3.5 for intra-cardiac structures and >3.0 for coronary arteries. Intra-cardiac morphology and vascular anatomy were well visualized with good interobserver agreement (*r* = 0.46). Correspondence between the findings on MUSIC, surgery, correlative imaging and autopsy was excellent. The average clinical impact score was 4.2 ± 0.9. In five patients with discordant findings on echo/MUSIC (*n* = 5) and catheter angiography/MUSIC (*n* = 1), findings on FE-MUSIC were shown to be accurate at autopsy (*n* = 1) and surgery (*n* = 4). The decision to undertake biventricular vs univentricular repair was amended in 2 patients based on FE-MUSIC findings. Plans for surgical approaches which would have involved circulatory arrest were amended in two of 28 surgical cases. In all 28 cases requiring procedural intervention, FE-MUSIC provided accurate dynamic 3D roadmaps and more confident risk-to-benefit assessments for proposed interventions.

**Conclusions:**

FE-MUSIC CMR has high clinical impact by providing accurate, high quality, simple and safe dynamic 3D imaging of cardiac and vascular anatomy in neonates and infants with CHD. The findings influenced patient management in a positive manner.

**Electronic supplementary material:**

The online version of this article (doi:10.1186/s12968-017-0352-8) contains supplementary material, which is available to authorized users.

## Background

Within the past decade, advances in cardiovascular imaging and therapeutic interventions have improved the care of newborns with congenital heart disease (CHD). Severe congenital anomalies are nowadays often detected with fetal echocardiography during gestation such that elective deliveries can be planned at centers of excellence. While echocardiography is clearly the first line of imaging in neonates, even in the best of hands there may remain unanswered questions. In these cases, cardiovascular magnetic resonance (CMR) has emerged as a second line technique that does not involve ionizing radiation. Further, in small children with complex CHD and multisystem involvement, CMR holds promise for comprehensive pre-surgical evaluation with catheter angiography reserved for select indications and image-guided interventions.

CMR in pediatric CHD however, is highly specialized and individualized as almost all sequences require adaptation for body size, heart rate and specific clinical questions. Compared to older children, the requirement for spatial resolution is more stringent. Further, CMR exams in this population should be brief due to the high acuity of many cases and to minimize time outside of the neonatal intensive care unit (NICU). Conventional CMR techniques for pediatric CHD typically involve a timed bolus of a gadolinium based contrast agent (GBCA) for 3D vascular evaluation, in addition to multiple 2D breath hold cine acquisitions in customized orientations. A 4D imaging approach can potentially combine the best of both worlds, providing high resolution 3D anatomy and an unlimited number of 2D cine planes for reconstruction in arbitrary orientations.

Recently, a 4D Multiphase Steady-state Imaging with Contrast (MUSIC) CMR technique was introduced, which generates sub-millimeter, isotropic 3D voxels over multiple phases of the cardiac cycle [1]. MUSIC data are acquired without breath holding during continuous positive pressure ventilation, and the airway pressure signal is used for respiratory gating. Cardiac gating is implemented using the ECG and a stable blood pool signal is assured by imaging during the steady state distribution of ferumoxytol (Feraheme®, AMAG, Lexington, MA). With a total scan time of 7–10 minutes, MUSIC offers the potential for rapid, simple, safe and versatile mapping of complex cardiovascular anatomy with dynamic resolution previously not available.

In this study, we aim to evaluate the diagnostic quality and performance of ferumoxytol (FE) enhanced MUSIC CMR in a cohort of neonates and infants with CHD and to assess its impact on patient management.

## Methods

This prospective study was approved by our Institutional Review Board and was compliant with the Health Insurance Portability and Accountability Act. Written informed consent was obtained from legal guardians of all subjects. Forty consecutive neonates and infants with CHD (age range 2 days to 2 years; 21 females; weight range 1 to 13 kg) undergoing an FE-CMR from 2013 to 2016 were enrolled, including two recent subjects enrolled under IND #129441 (Clinicaltrials.gov NCT02752191). No patients were excluded. Primary study indications were: 1) assessment of vascular anatomy (*n* = 20), 2) intra-cardiac anatomy (*n* = 17), 3) pre-interventional or surgical planning (*n* = 16). Primary diagnoses are outlined in Additional file 1: Table S1.

In patients whose blood gas status was felt sufficiently stable for safe breath holding by attending neonatologists or anesthesiologists, breath-held 3D FE-CMRA (cardiovascular magnetic resonance angiography) was performed as a standard of care reference for comparison of image quality with MUSIC.

### MR Acquisition

All neonates and infants were examined during continuous positive pressure ventilation as is standard at our institution and at many centers performing neonatal CMR [2, 3]. Patients were transported directly to the CMR suites, already intubated and sedated. Sedation management and physiologic monitoring have been described previously [4] and typically included IV fentanyl and rocuronium. Continuous monitoring of heart rate, blood pressure, pulse oximetry, and end-tidal CO_2_ was performed and recorded in all cases.

All studies were performed on a clinical 32-channel 3.0 T system (Magnetom TIM Trio, Siemens Medical Solutions). Phased-array multi-element coils were used for signal reception, in configurations based on body size. For children weighing less than 2 kg, a 16-channel adult extremity (knee) coil was employed. For children weighing 2 kg or more, a combination of head-neck (posterior elements) and small flex coil (anterior elements) was used. Imaging parameters for breath-held FE-CMRA were: repetition time/echo time (TR/TE) 2.9/0.9 ms; flip angle 15-17°; in-plane resolution 0.9–1.2 mm; slice thickness 0.9–1.1 mm; GRAPPA acceleration 3×–4×; 75% partial Fourier acquisition in both phase encoding directions. FE-MUSIC was acquired during continuous ventilation using the airway pressure signal for respiratory gating [1]. No adjustments were made to the ventilatory frequency, amplitude or waveform to maximize gating efficiency and the default settings were employed. Technical parameters for FE-MUSIC were: TR/TE 2.9/0.9 ms; flip angle, 25°; 3D isotropic (non-interpolated) resolution 0.6–0.9 mm; GRAPPA 2×–3×; 75% partial Fourier in both phase encoding and partition encoding directions. The pressure waveform from the endotracheal tube was input into the physiological monitoring unit of the CMR scanner and served as a surrogate respiratory gating signal. An empiric respiratory gate time delay of three cardiac segments (~200 msec) was used to account for the temporal phase lag between the upper airway pressure wave and resulting diaphragmatic movement. A 50% threshold of the pressure peak defined the acceptance range for the respiratory gating.

A total of 4 mg/kg (elemental iron per kg of body weight) of the stock ferumoxytol formulation was diluted with normal saline by 8×–10× based on patient size [4]. For first pass imaging (*n* = 15), half of the diluted ferumoxytol solution (2 mg/kg) was infused over 15 s [5]. The injection duration was 75% of the acquisition time window [4]. Subsequently, the remaining half of the diluted ferumoxytol was administered over 30 s to provide a total dose of 4 mg/kg for steady state imaging. To comply with a warning issued by U.S. Food and Drug Administration (FDA) in March 2015 [6], our protocol was amended to give ferumoxytol only by slow infusion at a rate of 0.8 mg/kg/min. Therefore, in 25 of 40 subjects who received ferumoxytol, only steady state imaging was performed.

### Image analysis

Two experienced CMR readers independently scored the images. FE-MUSIC images were reviewed using ‘multiplanar reconstruction (MPR) cine mode’ on a Mac-OsiriX workstation (OsiriX MD version 6.5, Pixmeo, Switzerland), which enables interactive dynamic, multiphase interrogation of arbitrary imaging planes until the optimal plane for visualization is chosen. For comparison with the single phase, breath-held FE-CMRA images, each reader was free to choose a preferred single 3D phase of the FE-MUSIC images for each of the intra-cardiac structures (valves, cardiac chambers), ventricular outflow tracts, and named vascular segments, including the proximal coronary arteries, using a four-point scale [7] (Additional file 1: Table S1). For coronary artery visualization, the optimal cardiac phase and plane for coronary visualization was interrogated. Based on border definition, image contrast, and presence of artifacts, scores of 1 or 2 were considered non-diagnostic whereas scores of 3 or 4 were considered diagnostic. MUSIC findings were correlated with surgical reports (*n* = 28), catheterization data (*n* = 14), cardiac CT data (*n* = 4), and autopsy findings (*n* = 1). The signal-to-noise ratio (SNR) was calculated as the ratio of the mean luminal signal intensity (SI) divided by the standard deviation of noise and was further divided by 1.53 to adjust for Rayleigh noise distribution [8]. The contrast-to-noise ratio (CNR) was calculated as the difference between the SNR of the lumen and SNR of nearby muscle tissue. Noise was defined by the standard deviation in regions of interest of air within the imaging field of view.

### Clinical impact

The impact of FE-MUSIC on overall diagnosis and patient management for each case was scored in consensus with collaborating surgeons and cardiologists. Five key measures of added value were assessed: 1) change in overall surgical management, 2) change in surgical approach, 3) reduction in the need for diagnostic catheterization, 4) improved assessment of risk-to-benefit for planned intervention and discussion with parents, 5) accurate pre-procedural roadmap.

### Statistical analysis

Statistical analysis was performed using MedCalc 12.0.1.0 (Mariakerke, Belgium). Continuous data were summarized as mean ± standard deviation or as mean and interquartile. Categorical data were summarized as absolute values and frequencies. The Wilcoxon rank sum test was used to compare the image quality scores between breath-held FE-CMRA and FE-MUSIC. Pearson’s correlation coefficient was used to determine inter-observer correlation. Analysis of variance (ANOVA) for repeated measurements was used to determine the statistical significance of temporal changes in mean heart rate, blood pressure, pulse oximetry (SpO_2_), and end-tidal CO_2_. A p value < 0.05 is considered significant.

## Results

All 40 patients (neonates [*n* = 20, 2 to 25 days, 1–4 kg]; infants [*n* = 20, 1.2 months to 2 years, 2–13 kg]) underwent the ferumoxytol-enhanced exam safely and without any adverse events, including those who had bolus injection of ferumoxytol for breath held FE-CMRA. Total image acquisition time for FE-MUSIC ranged from 7 to 10 mins. The SNR and CNR of the FE-MUSIC images were 54 ± 21 and 38 ± 15, respectively. Clinical complexity and diagnoses of the patient cases are outlined in Additional file 1: Table S2. Heart rate, blood pressure, blood oxygenation and end tidal CO_2_ remained stable throughout the procedure and variations were not statistically significant (*p* > 0.05) (Additional file 1: Table S3).

### Image quality

Image quality scores are reported in Table 1. Respiratory gating efficiency for the MUSIC acquisition ranged from 45 to 58%. Of the 13 intra-cardiac structures and vascular segments evaluated, FE-MUSIC had an average image quality score greater than 3.5 in 12 structures and 3.3 for the coronary arteries. Based on a four-point scoring system, scores of 3 or greater indicate that all relevant cardiac and vascular structures within the imaged field of view were confidently evaluable on FE-MUSIC images. Inter-reader correlation of FE-MUSIC scores was higher (*r* = 0.46, 95% CI 0.37 to 0.55, *p* < 0.01) compared to FE-CMRA (*r* = 0.41, 95% CI 0.30 to 0.58, *p* < 0.01). In the subset of babies (*n* = 15) with both FE-CMRA and FE-MUSIC, FE-MUSIC images provided superior visualization of intra-cardiac anatomy and superior vascular definition (*p* < 0.001). For FE-CMRA images, the average image quality scores were <2.5 for all intra-cardiac structures, outflow tracts, and coronary arteries.

**Table 1:** Image quality score of FE-CMRA and FE-MUSIC

	FE-CMRA (*n* = 15)	FE-MUSIC (*n* = 40)	*P* value*
Right atrium	1.9 ± 0.4	3.7 ± 0.5	*p* < 0.001
Left atrium	2.0 ± 0.4	3.8 ± 0.4	*p* < 0.001
Right ventricle	2.0 ± 0.4	3.6 ± 0.5	*p* < 0.001
Left ventricle	2.0 ± 0.4	3.7 ± 0.5	*p* < 0.001
Interatrial septum	1.5 ± 0.6	3.8 ± 0.4	*p* < 0.001
Interventricular septum	2.2 ± 0.7	3.8 ± 0.4	*p* < 0.001
Tricuspid valve	1.4 ± 0.5	3.6 ± 0.5	*p* < 0.001
Mitral valve	1.4 ± 0.5	3.7 ± 0.4	*p* < 0.001
LVOT, aortic valve, and aortic root	2.0 ± 0.7	3.7 ± 0.5	*p* < 0.001
RVOT, pulmonary valve	1.9 ± 0.7	3.6 ± 0.5	*p* < 0.001
Main pulmonary artery and second order branches	2.7 ± 0.6	3.6 ± 0.6	*p* < 0.001
Proximal ascending aorta	2.8 ± 0.6	3.7 ± 0.6	*p* < 0.001
Coronaries	1.2 ± 0.5	3.3 ± 1.0	*p* < 0.001

*LVOT* left ventricular outflow tract, *PV* pulmonic valve, *RVOT* right ventricular outflow tract

*P values reflect comparisons between average image quality scores for FE-CMRA and FE-MUSIC using the Wilcoxon rank sum test

**Fig. 1:**
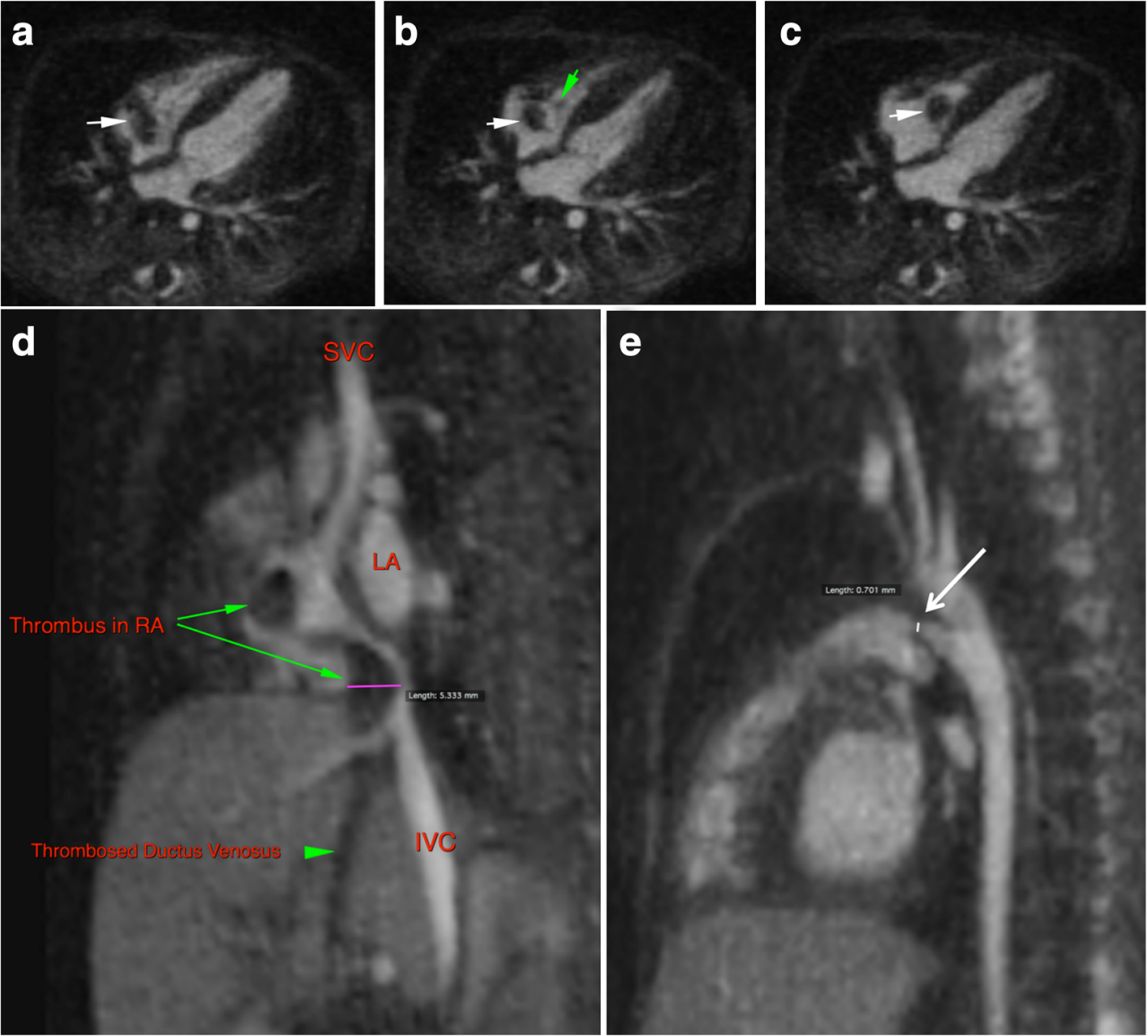
FE-MUSIC images of a twenty-one-day old boy (1.1 kg) with a patent ductus arteriosus (**a**-**e**). A mobile right atrial mass of uncertain etiology was noted on echocardiography after birth. Three of 8 frames from FE-MUSIC show a large mobile mass (*white arrow*) in the right atrium (RA) abutting the tricuspid annulus and valve leaflets (*green arrow*). The mass has to-and-fro motion and connects via a thin stalk to thrombus (**d**, Additional file 3: online video 1a) in the inferior vena cava, which in turn, is continuous with thrombus in the ductus venosus originating from an infected umbilical vein catheter. A patent ductus arteriosus measuring 700 microns at its waist is shown bridging the pulmonary artery and descending aorta in the FE-MUSIC image (**e**, Additional file 3: online video 1b)

**Fig. 2:**
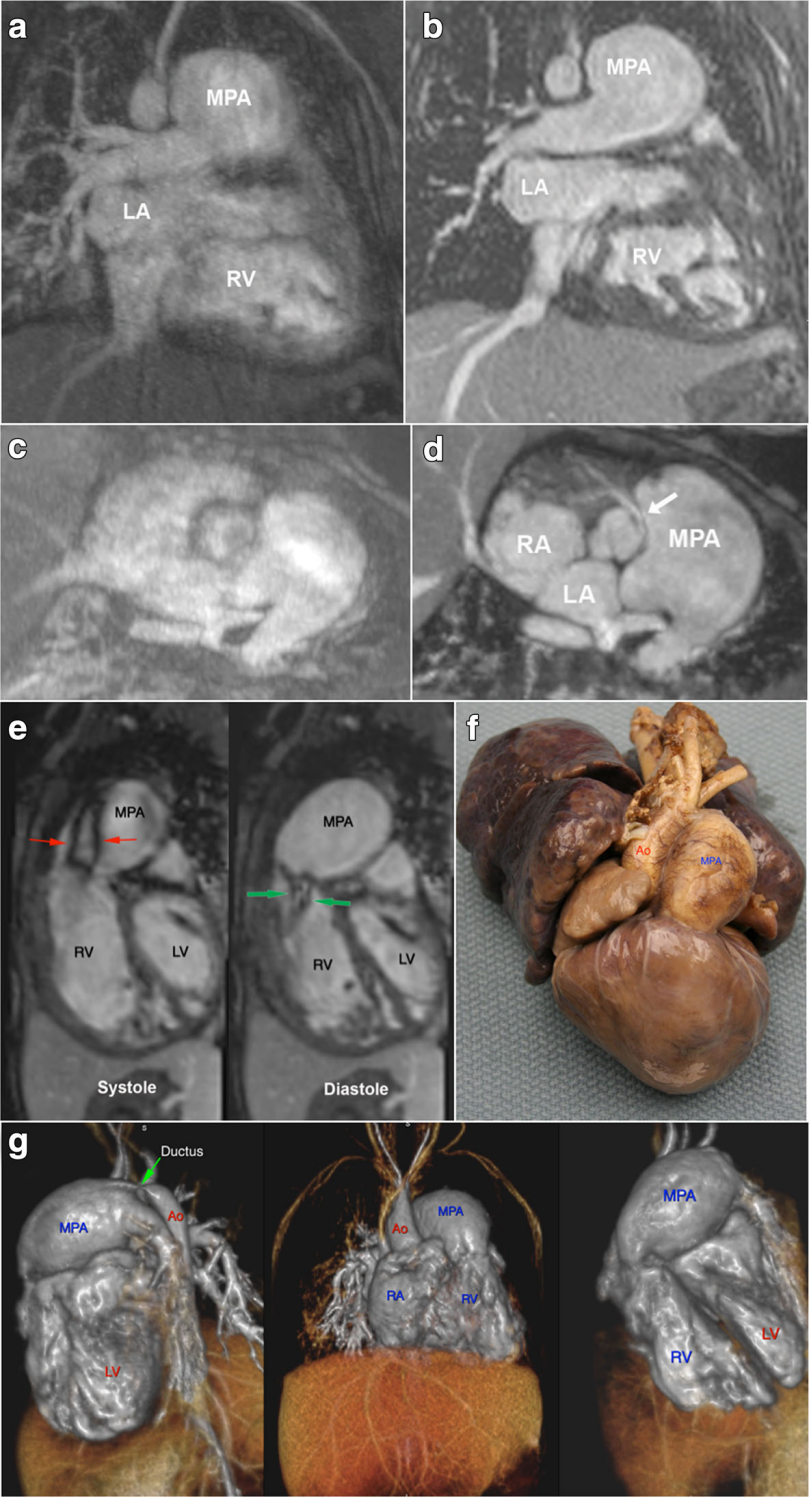
Correlative FE-MUSIC and autopsy findings of a premature newborn girl (1.6 kg) with severe pulmonary regurgitation, dilated main pulmonary artery, right ventricular hypertrophy, and anomalous right coronary artery are shown. Breath held FE-CMRA (**a**, **c**) shows blurred cardiac borders, poor definition of RV trabeculae (scored 1) and mild blurring of the severely dilated main pulmonary artery (scored 3). FE-MUSIC (**b**, **d**) shows defined cardiac chambers with hypertrophied and trabeculated right ventricle (RV, scored 4) and interatrial septum (scored 4). The right coronary artery has an anomalous origin from the left coronary cusp and an inter-arterial course (*white arrow* in **d**, scored 3). An ejection flow jet (*red arrows*) and a regurgitant flow jet (*green arrows*) are visualized in systolic and diastolic FE-MUSIC frames (**e**) respectively. Autopsy findings (**f**) show full agreement with volume-rendered FE-MUSIC images (**g**). Ao, aorta; MPA, main pulmonary artery; RA, right atrium; RV, right ventricle

Figures 1 and 2 and Additional file 2: Figure S1 provide a range of comparative examples of FE-CMRA and FE-MUSIC image quality. Videos are provided as online files to highlight the incremental benefit of dynamic multiphase display. The online videos illustrate clear definition of valve leaflets and the dynamic relationship of great vessels to intracardiac structures over multiple phases of the cardiac cycle. Using the interactive cine mode of multiplanar reformats, arbitrary planes of the beating heart could be interrogated. This latter feature facilitated more confident visualization of cardiac chambers and coronary anatomy, provided a roadmap for surgical planning, and enabled more confident risk-to-benefit assessment. The quality of FE-MUSIC is exemplified by illustrative examples of coronary anatomy in neonates and infants whose heart rate are physiologically tachycardic and clear visualization requires high spatial resolution (Additional file 2: Figure S1, Fig. 2c-d, Fig. 4b-c, Fig. 6, Additional file 3: online video 6a). In an unstable 1.1 kg patient with a tiny 700 micron patent ductus arteriosus (PDA), acidosis, and a heart rate of 160–180 bpm (Fig. 1), multi-planar thin slice (0.8 mm) cine reconstruction of the FE-MUSIC data showed the origin, nature and extent of thrombus from an infected umbilical vein catheter to the right atrium. Successful and complete surgical thrombectomy was performed without further imaging.

### Correlation of MUSIC Findings with other Modalities

MUSIC findings correlated well with angiographic data (*n* = 14), cardiac CT (*n* = 4), and surgical reports (*n* = 28). Autopsy findings were available for one neonate (Fig. 2). In five patients with discordant findings (echo and MUSIC [*n* = 5]; catheter angiography and MUSIC [*n* = 1]), the findings on FE-MUSIC were shown to be accurate at autopsy (*n* = 1) or surgery (*n* = 4). Images in Fig. 2 are of a premature neonate weighing 1.5 kg who underwent CMR following echo and angiography to further clarify vascular and intra-cardiac anatomy. The patient had a heart rate of 160–170 beats per minute (bpm). FE-MUSIC showed a dysplastic pulmonic valve with right ventricular enlargement, an aneurysmal pulmonary artery and severe pulmonary regurgitation. An anomalous right coronary artery was incidentally noted on FE-MUSIC (Fig. 2d), which had not been evident on echo or catheterization nor well-visualized on FE-CMRA (Fig. 2c). Comparative breath-held FE-CMRA produced poor definition of intra-cardiac (Fig. 2a) and coronary anatomy (Fig. 2c). Autopsy examination confirmed the aforementioned findings from FE-MUSIC, in addition to a small PDA. Because of the neonate’s poor renal function and critically ill status, very little iodinated contrast could be used during catheterization. Thus, there was poor contrast opacification of the high capacity right ventricular outflow tract, where suboptimal visualization suggested an aortopulmonary window versus a large PDA. FE-MUSIC clarified and reconciled the findings between echo and catheterization, and definitively depicted the vascular and intra-cardiac anatomy.

### Impact of FE-MUSIC on Patient Management

**Fig. 3:**
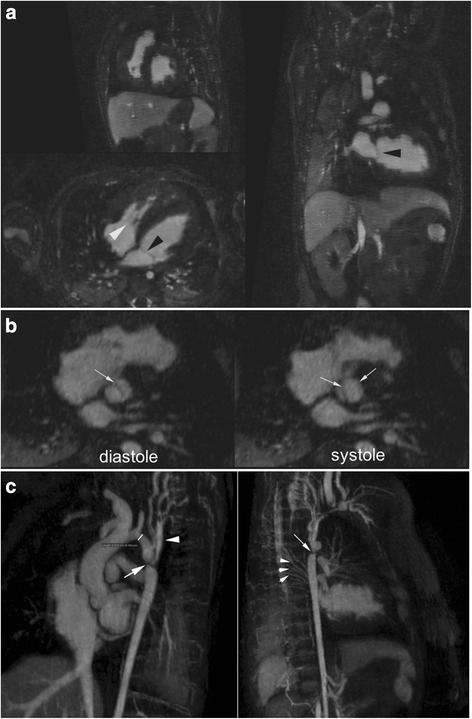
Multiplanar reformat MUSIC images of a 1-month old boy infant (4.4 kg) with biventricular hypertrophy (**a**), bicuspid aortic valve (**b**), and critical aortic coarctation (**c**) are shown. *Black arrowheads* (**a**) point to thin mitral valve leaflets. Tricuspid valve leaflets and chordae are well characterized (**a**, *white arrowhead*). Bicuspid aortic valve leaflets (**b**, *white arrows*) demonstrate good excursion throughout the cardiac cycle. The transverse aortic arch (**c**, *white line*) is hypoplastic (0.32 cm). Critical aortic coarctation (**c**, *white arrow*) along with collaterals (**c**, *white arrowheads*) and their dynamic relationship to intracardiac anatomy are well characterized (Additional file 3: Online video 3b). Vessels and intracardiac borders are sharp. There is moderately reduced left ventricular systolic function (Additional file 3: Online video 3b). Turbulent flow through the bicuspid valve and minimal flow through the coarctation are demonstrated in Additional file 3: Online video 3b. FE-MUSIC CMR was ordered to define vascular structures prior to surgery and to delineate the etiology for reduced left ventricular systolic function. Because of the severe coarctation, arch hypoplasia, and reduced left ventricular systolic function, the patient underwent repair of the coarctation and arch augmentation. The global LV hypokinesis and systolic function improved after surgical intervention. The arch anatomy was unclear on echo and the FE-MUSIC findings changed the surgical plan as well as facilitated discussion with parents regarding the overall management plan

**Fig. 4:**
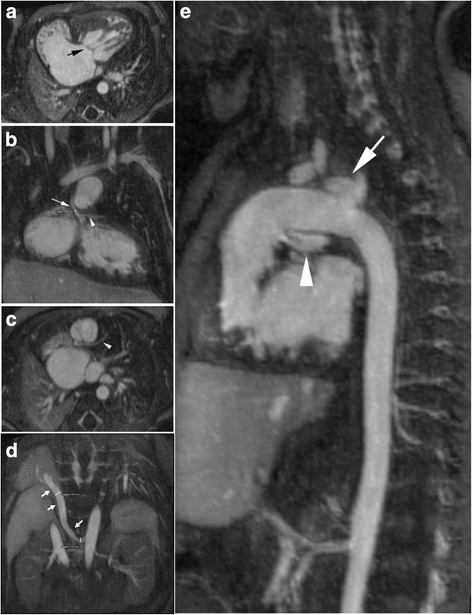
A 10-day old neonate (3.6 kg) with hypoplastic left heart syndrome (HLHS) who was referred for FE-MUSIC CMR to assess pulmonary vein stenosis and to delineate intra-cardiac and extracardiac vascular anatomy prior to defining a surgical approach. His heart rate ranged from 126 to 140 beats per minute. HLHS with predominant right heart anatomy (**a**, multiplanar reformat) and common atrioventricular valve (*black arrow*) were confirmed. There were large pulmonary arteries and a diminutive aortic root (**b**, *white arrow*; aortic annulus 2.5 mm, sinotubular junction 1.2 mm) with the left main coronary artery (white arrowhead) coming off the aortic sinus. The left anterior descending artery courses between the RVOT and ventricle (**c**, white arrowhead). Large APCs (**d** and Additional file 3: Online video 4a-4b, *white arrows*) from the abdominal aorta were seen. The ductal arch (**e**) is continuous with the descending aorta. *White* arrow points to the innominate artery and *white arrowhead* points to the left pulmonary artery. No pulmonary vein stenosis. Based on the findings, the patient underwent occlusion of APCs and ductal stenting prior to proceeding with a hybrid Norwood and bilateral banding of the pulmonary arteries. Because MUSIC images provided a clear roadmap for surgery planning, our surgeons and cardiologists had a better sense of the child’s higher risk profile. MUSIC enhanced the risk discussion with the child’s parents. As a result, the decision was to palliate rather than pursue a staged operation

**Fig. 5:**
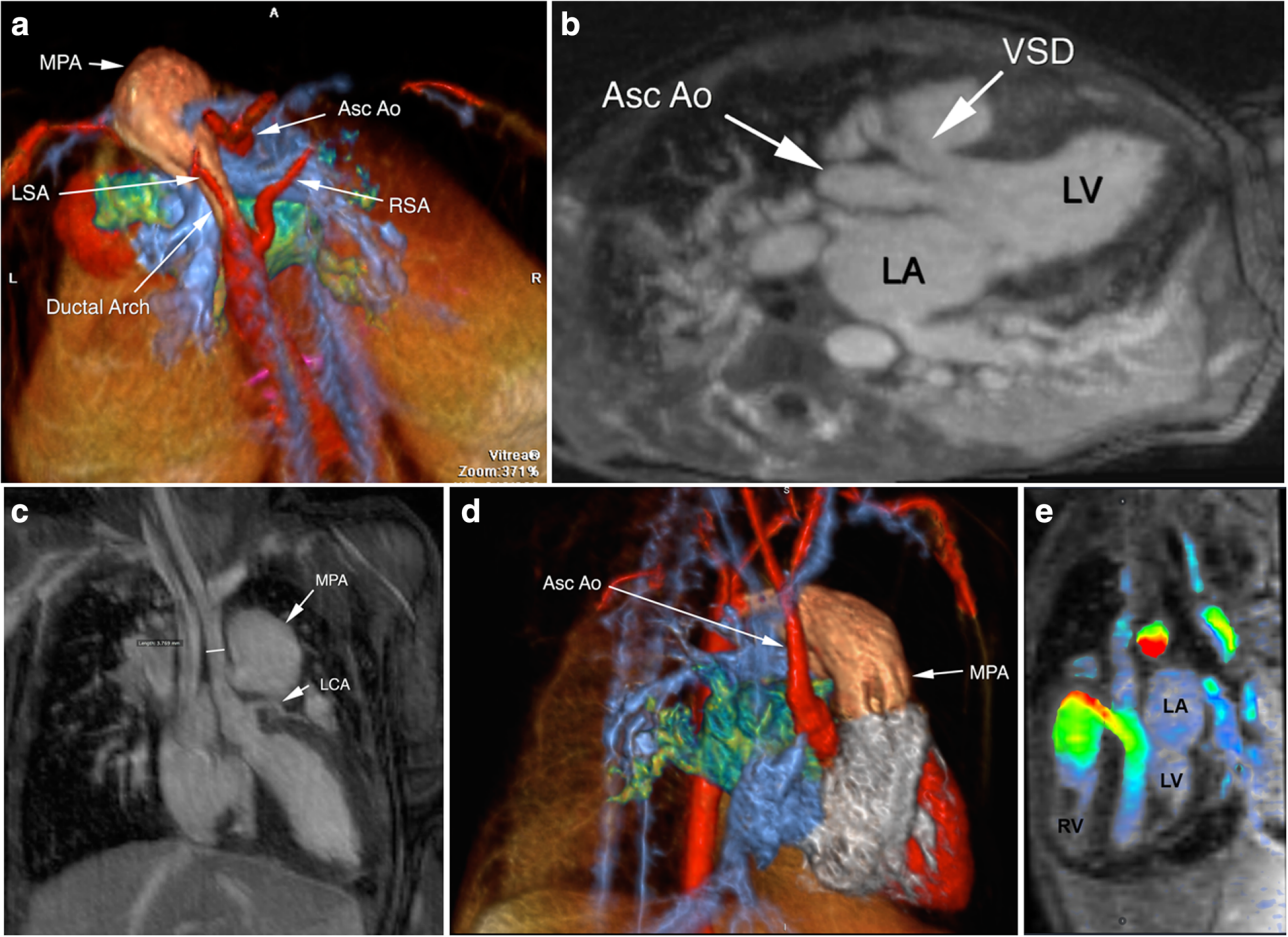
3D multiplanar reformat and color volume rendered MUSIC images of a 20-day old neonate (2.1 kg) with interrupted ascending aorta (**a**, bird’s eye view, Asc Ao) and ventricular septal defect (VSD, **b**) are shown. 4D color volume rendered MUSIC images are available as Additional file 3: Online video 5a. Relationships between the large main pulmonary artery (MPA), hypoplastic ascending aorta (3.8 mm), ductal arch, and intracardiac structures are depicted in **c** and **d**). Their 4D dynamic relationships are exemplified in Additional file 3: Online video 5a. The proximal course of the left coronary artery (LCA) is well visualized (**c**). FE-MUSIC CMR was obtained to clarify extra-cardiac vessels and intra-cardiac anatomy. Her heart rate ranged between 137 and 184 beats per minute. Clear definition of intra-cardiac anatomy along with findings of predominant flow through the VSD (**e**, Additional file 3: Online video 5b-c) resulted in the patient undergoing biventricular rather than single ventricle repair. LA, left atrium; LSA, left subclavian artery; LV, left ventricle; RSA, right subclavian artery

**Fig. 6:**
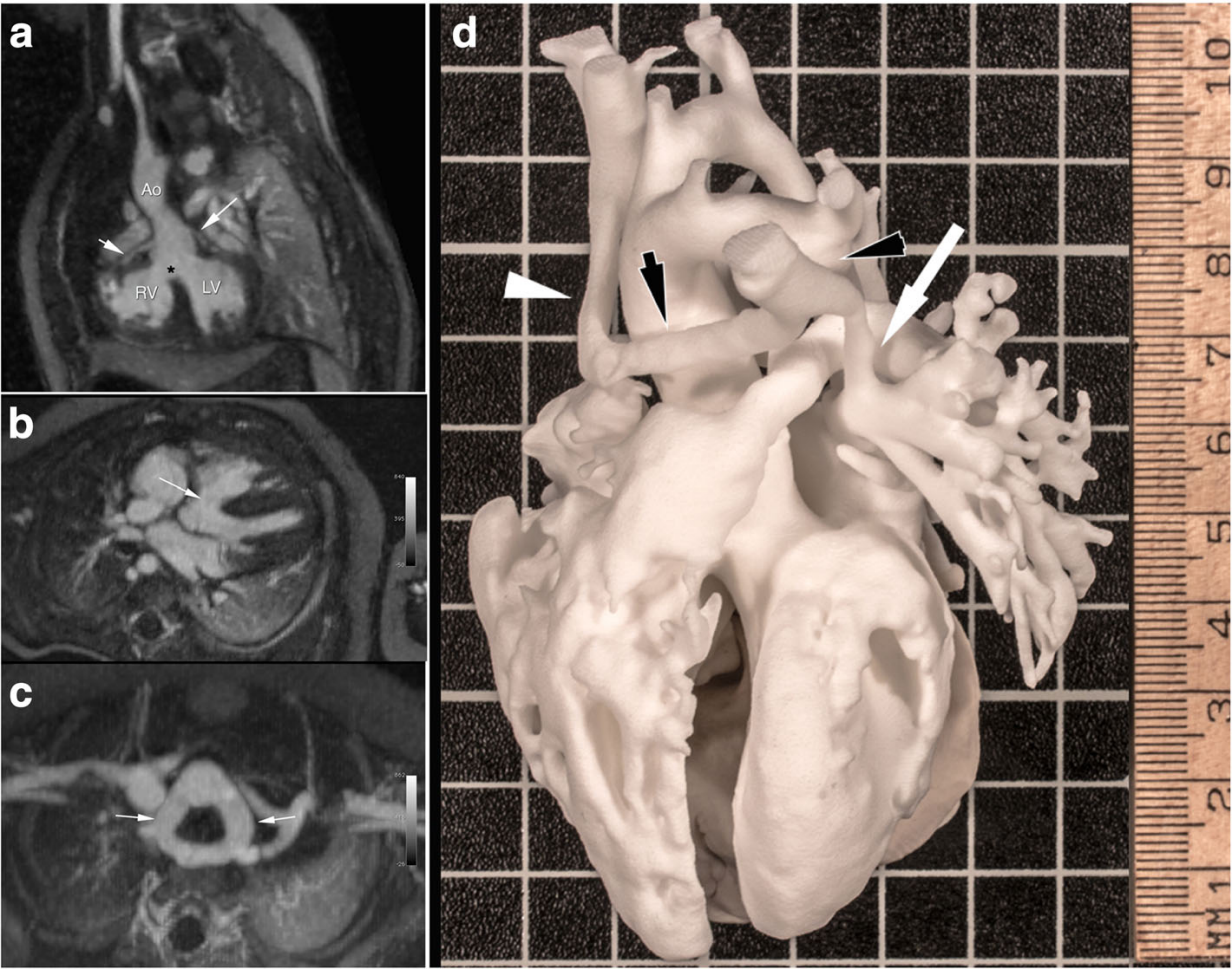
Multiplanar reformats of FE-MUSIC in a 3-month old girl (7.7 kg) with Tetralogy of Fallot (ToF) and a double aortic arch. Characteristic features of ToF (**a**, Additional file 3: Online video 6a) including right ventricular (RV) hypertrophy with dynamic RV outflow tract obstruction, an overriding aorta, and a perimembranous ventricular septal defect (*black asterisk*) are clearly visualized on dynamic review. Both proximal courses of the left and right coronary arteries (**a**, *white arrow*; Additional file 3: Online video 6a) are also well visualized; the distal right coronary artery can be seen coursing along the right ventricle. Additional file 3: Online video 6a exemplifies the value of dynamic, multiphase imaging in the setting of coronary visualization. The large ventricular septal defect (**a**, *black asterisk*; **b**, *white arrow*) and the complete vascular ring from a double aortic arch (**c**, *white arrows*) are clearly delineated. There is no dynamic compression of the trachea. Colorized volume rendered cine MUSIC images (Additional file 3: Online video 6b) illustrate the dynamic complex extra-cardiac vascular anatomy and its relationship to intra-cardiac structures, which can be used to provide a more concrete image of the anatomic problem and explain a clearly complex case to parents and guardians (**d**, Additional file 3: Online video 6c). There is anomalous pulmonary venous drainage with the left innominate vein (*black arrow*, **d**) dipping inferiorly before joining the right innominate vein (*white arrowhead*, **d**) to form a right-sided superior vena cava. The left superior vertical vein (*black arrowhead*, **d**) joins the low bridging left innominate vein (*black arrow*, **d**) and the left superior pulmonary vein (*white arrow*, **d**), which forms the confluence of the superior pulmonary venous trunk (Additional file 3: Online video 6b, left panel). There is also a double aortic arch, which forms a complete vascular ring without tracheal compression (Additional file 3: Online video 6b, right panel). The FE-MUSIC data were further processed for 3D printing (photographed in **d**, Additional file 3: Online video 6c). The patient subsequently underwent successful surgical repair

In all cases, FE-MUSIC satisfactorily answered all clinical questions requested by referring surgeons or cardiologists and in many cases provided additional insight, which impacted the overall patient care plan. Table 2 provides sample cases highlighting the clinical impact of the FE-MUSIC findings on patient management. On a five-point scale (with one point for each key clinical outcome measure), the average clinical impact score was 4.2 ± 0.9. In all cases, results from FE-MUSIC informed the overall management of the patient and informed the risk-to-benefit evaluation for discussion with the patient’s parents regarding the treatment plan (Figs. 3, 4, 5 and 6). Of the 28 cases where management may have needed additional assessment by catheterization, further diagnostic evaluation was avoided in 13 patients because relationships between vascular anatomy and its relationship to intracardiac morphology were well visualized on FE-MUSIC imaging. Supplemental information regarding flow patterns and volume was available as a part of the entire clinical CMR exam (Fig. 3, Additional file 3: online video 3b; Fig. 5, Additional file 3: online video 5b and 5c). One diagnostic cath was performed at an outside facility prior to CMR. Of the remaining 14 cases that had catheterization, three patients had necessary pre-Glenn angiograms and 11 underwent catheterization for therapeutic purposes (four of which were transcatheter interventions that were in lieu of high risk surgery). Sixty-eight percent of patients (*n* = 16 neonates; *n* = 12 infants) had successful surgical correction or palliation of congenital anomalies. Circulatory arrest was avoided in 2 surgical cases. In all cases requiring intervention, multiphase assessment of vessel size and dynamic 3D imaging of intra- and extra-cardiac structures facilitated procedural planning by decreasing projected operative time as well as informing more confident assessment of the true risks and benefits of the proposed procedure. For example, in the case involving a 10-day neonate with hypoplastic left heart syndrome (Fig. 4, Additional file 3: online video 4a-4b), identification of large aortopulmonary collaterals from the abdominal aorta altered the course of management and surgical approach. In another patient, clarification of the complex anatomy with 3D volumetric reconstructions (Fig. 6) and 3D printing of FE-MUSIC images (Fig. 6d, Additional file 3: online video 6b-6c) helped cardiologists to communicate with parents and allowed surgeons to visually describe the operative plan to them. Prior knowledge of potential procedural risks was felt to be of significant value when discussing treatment options with parents or guardians. Of those undergoing surgery, decisions about the optimal surgical approach were informed by visualization of dynamic cardiac anatomy shown with FE-MUSIC (Figs. 3, 4, 5 and 6).

**Table 2:** Case examples highlighting the clinical impact of FE-MUSIC CMR on the management of neonates and infants with congenital heart disease

Pt	Weight (kg)	Pre-MUSIC diagnosis	Post-MUSIC diagnosis	Management and impact on patient care
1	2.2^d^	TOF-PA, discontinuous PAs	TOF-PA, right-sided aortic arch with diminutive MPA (1.3 mm) and branch PAs, MAPCAs arising from LSCA & DAo	Balloon angioplasty of PAs, BT shunt deferred until patient is ~3 kg
2	2.6	TOF-PA, ?discontinuous PAs	TOF-PA with discontinuous PAs, MAPCAs supplied RPA, ductus/APCs from distal abdominal aorta supplies LPA	Unifocalization of PAs, patch angioplasty at small MPA/RPA juncture, and modified left BT shunt
3	3.3	D-TGA with VSD, double aortic arch, sub-PS/PS	D-TGA, VSD, double aortic arch with tracheo-esophageal compression, sub-PS/PS. Incomplete tracheal rings.	Underwent balloon atrial septostomy; subsequent staged surgery with modified right BT shunt, division of DAA
4	2.5^ac^	Hypoplastic aortic arch; VSD	Severe aortic arch hypoplasia, near IAA, VSD, normal LV volume/size	VSD closure and aortic arch augmentation
5	3.5	TOF-PS	TOF-PS, hypoplastic MPA continuing as RPA. No APCs. LPA comes from transverse aorta. LCA originates from LCC and courses between RPA and aorta without compression	Staged unifocalization of LPA aided by visualization of unconventional coronary course
6	1.5^ab^	PV dysplasia, moderate PR, bicuspid AV, severe RVH, ?AP window	PV dysplasia, severe PR, anomalous RCA from LCC with acute anterior angulation, severe RVH. Left-sided aortic arch. No AP window seen	Unsuccessful PDA closure. Patient expired prior to surgery. Autopsy confirmed MUSIC findings.
7	3.6	HLHS,?pulmonary vein stenosis	HLHS, large PAs, no pulmonary vein stenosis. Preserved ventricular function. Large APC from DAo	Occlusion of APC and ductal stenting prior to hybrid Norwood with bilateral banding of PAs
8	2.8^d^	SV/heterotaxy with PA, ?APCs, PAPVR vs TAPVR	SV/heterotaxy, TAPVR, hypoplastic PAs with MAPCAs	Ductal stenting; No surgery
9	1.5	Parachute MV, bicuspid AV with AS, aortic coarctation	Parachute MV, hypoplastic LV, bicuspid AV with severe AS, hypoplastic aortic arch	ABVP and BAS, Subsequent aortic arch repair
10	2.6	TOF-PA	TOF-PA, confluent branch pulmonary arteries. No MAPCAs.	BT shunt, ductal ligation
11	2.4^d^	TOF-PA, Unclear PAs anatomy	TOF-PA, absent MPA, tortuous L/RPA, MAPCAs from proximal left vertebral artery to LPA, MAPCAs from RSCA to RPA. Severe RPA hypoplasia (1.7 mm)	Left subclavian collateral stenting. Small PAs size led to stenting and deferring unifocalization
12	4.2^a^	VSD, aortic arch and branching not well seen	VSD, vascular ring with right aortic arch and aberrant left brachiocephalic artery coursing posteriorly, inferiorly behind esophagus and trachea. No tracheal compression.	VSD closure, division of vascular ring. Extracardiac characterization of vascular ring’s unusual course facilitated surgical planning; surgery occurred earlier because of VSD
13	3.2a	Double aortic arch, large VSD	IAA with LPA & LSCA arising from left branch of hypoplastic AA, large VSD	VSD closure, IAA repair, LPA reimplantation rather than ring division
14	2.3	PAPVR, aortic arch hypoplasia	Scimitar syndrome with right-sided pulmonary sequestration; aortic arch hypoplasia	Occlusion of APCs, Surgical aortic arch repair
15	3.1	TOF-PA, LPA not well seen	TOF-PA, confluent branch PAs with discrete LPA stenosis, no MAPCAs	Surgery rather than watchful waiting. BT shunt with plasty of PAs rather than shunt only.
16	2.6	TOF-PA, LPA not well seen, ?APCs	TOF-PA, severe LPA stenosis, no MAPCAs	BT shunt with LPA plasty rather than watchful waiting
17	3.5^a^	Hypoplastic aortic arch	Double aortic arch forming complete vascular ring without tracheal or esophageal compression	Division of vascular ring rather than coarctation repair
18	2.1^c^	IAA/VSD, large PDA, hypoplastic bicuspid AV	IAA/VSD, large PDA, hypoplastic bicuspid AV; predominant flow thru VSD determined final surgical decision	Rastelli-type VSD closure with RV- PA conduit, Damus-Kaye-Stansel arch reconstruction
19	12.7	TOF/PA s/p repair (RV-PA conduit, VSD closure), MAPCAs s/p coil occlusion, RPA stenting	TOF/PA s/p unifocalization. No significant APCs. Findings of markedly diminished perfusion and arterial vascularity in the left lung base along with diminutive and pruned PAs to the LLL as well as dynamic compression of the LIPV determined surgical course	RV to pulmonary artery conduit replacement, aortic homograft, RPA stent removal, LPA repair
20	7.7	TOF/PS, double aortic arch with vascular ring, PAPVR	TOF/PS, double aortic arch with vascular ring. No compression of airways. 3D visualization of the PAPVR (left superior vertical vein joining the LSPV to the left innominate vein/subclavian vein junction) facilitated surgical approach and planning.	TOF repair, division of vascular ring, ligation of levoatrial cardinal vein

^a^Discordant echo/MUSIC findings (*n* = 5)

^b^Discordant catheterization/MUSIC findings (*n* = 1)

^c^Change from single ventricle to biventricular repair or vice-versa (*n* = 2)

^d^Percutaneous transcatheter intervention in lieu of immediate high risk cardiothoracic surgery (*n* = 3)

*AA* ascending aorta, *ABVP* aortic balloon valvuloplasty, *ASD* atrial septal defect, *AV* aortic valve, *BAS* balloon atrial septostomy, *BT* blalock-taussig, *DAo* descending aorta, *IAA* interrupted aortic arch, *LCA* left coronary artery, *L* (*R*) *PA* left (right) pulmonary artery, *L* (*R*) (*I*) (*S*) *PV* left (right) (inferior) (superior) pulmonary vein, *L* (*R*) *SCA* left (right) subclavian artery, *LV* left ventricle, *MAPCAs* major aortopulmonary collateral arteries, *MPA* main pulmonary artery, *MV* mitral valve, *NA* not applicable, *PA* pulmonary atresia, *PAPVR* partial anomalous pulmonary venous return, *PA* pulmonary atresia, *PAs* pulmonary arteries, *PDA* patent ductus arteriosus, *PFO* patent foramen ovale, *PH* pulmonary hypertension, *PV* pulmonic valve, *RCA* right coronary artery, *RV* right ventricle, *SV* single ventricle, *TAPVR* total anomalous pulmonary venous return, *TGA* transposition of the great arteries, *TOF* tetralogy of fallot, *VSD* ventricular septal defect

## Discussion

Our results affirm that FE-MUSIC provided detailed and reliable multiphase 3D visualization of intra-cardiac and extra-cardiac vascular anatomy in small babies. We also demonstrate that findings on MUSIC images had high clinical impact on the care of neonates and infants with CHD. FE-MUSIC represents a promising step towards practical and simplified image acquisition for combined assessment of dynamic cardiac and vascular anatomy in this complex patient group. Once the imaging volume is prescribed, no further interaction is required of the machine operator. FE-MUSIC images were immediately available for interrogation by interpreting physicians without additional post-processing. All relevant analyses and desired reconstructions were performed using a commercially available software with 4D capability. FE-MUSIC added clinical value by defining relevant dynamic anatomy clearly, providing detailed diagnostic data and procedural roadmaps, which informed decision-making, procedural planning and assessment of risks and benefits. Because no ionizing radiation is involved, CMR aligns well with the Image Gently® campaign to explore radiation-free imaging alternatives in children [9].

FE-MUSIC leverages reliable cardio-respiratory gating and ferumoxytol enhancement at 3.0 T to generate 4D images of the beating heart with true isotropic voxel dimensions of 0.6–0.9 mm [1]. This was accomplished by exploiting the high relaxivity and stable blood-pool concentration of ferumoxytol for steady state imaging, combined with regular airway pressure and ECG signals for optimal cardio-respiratory gating. Together, these strategies mitigate both respiratory and cardiac motion artifact and enable permanent and retrospective interrogation of cine images in any plane. Moreover, insofar as the respiratory waveform and heart rate remain regular and the blood concentration of ferumoxytol remains stable, FE-MUSIC can in principle expand to a high limit in both spatial and temporal resolution, overcoming many of the inherent challenges associated with CMR in pediatric CHD. These inherent challenges include small body size, high heart rates, immature renal function, need for repeated apnea, prolonged examination time, and dedicated physician supervision.

Several possible approaches exist for CMR in small children. Breath held, cardiac gated 3D contrast enhanced CMRA with gadolinium is an option [10, 11], but image acquisition is limited to one or two cardiac phases. Further, the limited time window for breath holding sets a limit on spatial resolution, temporal resolution and SNR. With FE-MUSIC, spatial resolution, temporal resolution and SNR have a high upper bound because breath holding is not necessary and image acquisition spans several minutes. SNR is likely higher for MUSIC at 3.0 T than at 1.5 T, although a comparison has not yet been performed in practice.

Others have described the use of 3D bSSFP [12–14] without contrast at 1.5 T. However, the requirement for high spatial resolution in small patients mandates a longer minimum TR per line for bSSFP which, when combined with the fast blood flow in neonates, predisposes to troublesome off-resonance artifact [15]. At 3.0 T, this phenomenon becomes even more problematic. On the other hand, the spoiled gradient echo acquisition in FE-MUSIC is insensitive to off-resonance effects at both field strengths and pulsatility artifacts are mitigated through gated, multiphase acquisitions. Although 3D respiratory navigator-gated and ECG-triggered IR-FLASH (inversion recovery - fast low-angle shot) with gadofosveset trisodium (Ablavar®, Lantheus Medical Imaging, MA) [16] has been described in CHD imaging, this technique produces only a single cardiac phase. FE-MUSIC provides a permanent 4D archive for reconstruction of cardiac-phase resolved images into any imaging plane, such that the requirement to acquire customized or unusual planes at the time of the study is obviated.

4D flow techniques represent another approach to image acquisition [17–19] in CHD and supplemental information on blood flow can be very helpful. However, with current 4D flow acquisitions, the requisite scan time needed to achieve the same spatial and temporal resolution as 4D MUSIC is greater by a factor of six or more, such that spatial and temporal resolution is usually dialed back with 4D flow [17]. Strategies for accelerated image acquisition are constantly in evolution and these will no doubt improve the performance of 4D flow techniques. Similar acceleration approaches can also be applied to 4D MUSIC such that it will likely remain proportionately faster than 4D flow. In practice, both techniques are complementary and it should not be necessary to choose one over the other. Ferumoxytol supports higher CNR for 4D flow as it does for 4D MUSIC and it seems logical to acquire both sets of data sequentially and to overlay the 4D velocity fields onto the high resolution dynamic anatomy of 4D MUSIC. While evaluation of 4D flow was not the focus of this current work, our referring surgeons and cardiologists had access to 4D flow images in the course of clinical decision making. Systematic evaluation of accelerated, ferumoxytol-enhanced 4D MUSIC and 4D flow as a single comprehensive technique for imaging of CHD is the subject of ongoing work in our laboratory and others.

With modern CT technology, it has become possible to generate 4D datasets at increasingly lower radiation doses. However, the radiation dose with multi-phase CT is proportionately greater than with single phase and raises concerns in young children, especially those who will likely require follow up studies. Nonetheless, clinical decisions are best made for individual patients depending on the required information and where ultrafast, low dose CT scanning is available, this may prove adequate and appropriate for assessment of static anatomy such as the site and size of aorto-pulmonary collateral vessels.

Although no ionizing radiation is involved with CMR, repeated imaging of babies with traditional approaches poses lifelong gadolinium exposure risks. In this regard, ferumoxytol offers an attractive alternative to GBCAs. Moreover, with the recent discontinuation of gadofosveset trisodium (Ablavar®), no blood pool CMR contrast agent is available clinically. Although ferumoxytol is approved by the FDA for treatment of iron deficiency anemia in adults with chronic kidney disease [20], as a theranostic agent, ferumoxytol also has potential for high fidelity steady state blood pool imaging because of its long intravascular half-life and high r_1_ relaxivity [5, 21–26]. Following US FDA approval in 2009, ferumoxytol has been used off-label [21, 22] as an alternative to GBCAs in patients with renal impairment [5, 24, 25, 27, 28]. In addition to its unique properties, the elemental iron in ferumoxytol is incorporated into the hematopoietic pathway once the outer carbohydrate shell is degraded providing a therapeutic source of iron, unlike GBCAs which has been shown to accumulate in biologic tissue [29] after repeated exposure. In neonates and children, the therapeutic effects of ferumoxytol may also be favorable as iron deficiency anemia is common in this population [30]. However, in light of a recent FDA warning regarding the rare potential for fatal hypersensitivity reactions, the benefit to risk ratios must be carefully weighed. To date, no major adverse events have been associated with the diagnostic use of ferumoxytol at our institution [31] and others [32, 33]. Moreover, in children, and particularly neonates, serious allergic reactions of any type tend to be less common than in adults (3–5.8% in children vs 6–10% in adults) [34] potentially because of a less developed immune system [35, 36].

While it is incumbent on caregivers to offer the best available options to patients and to inform management decisions thoughtfully, additional precautionary processes were implemented in our study. All of the patients had protected airways from the start of the procedure and hemodynamic monitoring was performed throughout the exam. While no adverse events occurred in our study, protocols were also in place to deal with them.

The focus on our current study is on the 4D MUSIC sequence in CHD for high resolution, dynamic imaging as opposed to the use of ferumoxytol as a contrast agent to replace gadolinium for all CMR applications. With this in mind, our study does have limitations. First, we did not directly compare GBCA-enhanced vs FE-CMRA in the same population for ethical and practical reasons. Second, we did not perform a systematic comparison between ferumoxytol and another blood pool contrast agent such as gadofosveset trisodium. Additionally, due to the retrospective and non-randomized nature of our study design, there is selection bias -- the neonates and infants studied generally represent the most complex cases and frequently with the highest degree of acuity. Because our study is retrospective and the investigation was implemented as a pragmatic clinical study in the context of routine clinical care, the surgical team was not blinded to the FE-MUSIC findings and an independent panel was not formed beforehand to assess the degree of divergence in the surgical plan pre and post FE-MUSIC results. Although preliminary work supports good agreement in volumetry between MUSIC and conventional 2D multi-slice SSFP cine imaging [1], effort is currently underway to systematically evaluate precision and reproducibility of volumetric measurements from 4D MUSIC. Our sample size is also modest. However, a multi-center clinical study is underway to systematically evaluate FE-MUSIC in CHD imaging and to adequately address these concerns. In spite of these limitations, our work demonstrates promising clinical evidence for using FE-MUSIC in neonates and infants with a wide range of CHD. Our findings also support the need for continued work towards the development and validation of streamlined, comprehensive techniques for imaging CHD.

## Conclusion

In neonates and infants with CHD, FE-MUSIC at 3.0 T depicts detailed, dynamic 4D anatomy reliably and with high clinical value, without the need for breath holding, contrast bolus timing or prescription of customized imaging planes. Further studies are warranted to assess the performance of FE-MUSIC across field strengths and imaging platforms.

## Additional files

Additional file 1: **Table S1.** Image quality scoring criteria. **Table S2.** Primary diagnoses of neonates and infants undergoing MUSIC CMR. **Table S3.** Hemodynamic Variation Before and After Injection of Ferumoxytol. (DOCX 47 kb)

Additional file 2: **Figure S1.** Multiplanar volume rendered MUSIC images from a 12-month old infant (11.4 kg) boy with a double aortic arch and complete vascular ring are shown in A-C. (DOCX 793 kb)

Additional file 3: Online videos. (ZIP 15120 kb)

## Abbreviations

CHD: Congenital heart disease
CMR: Cardiovascular magnetic resonance
CMRA: Cardiovascular magnetic resonance angiography
FDA: Food and Drug Administration
FE: Ferumoxytol enhanced
GBCA (s): Gadolinium based contrast agent (s)
MUSIC: Multiphase steady state imaging with contrast
NICU: Neonatal intensive care unit
SSFP: Steady state free precession
