# Supplementary material for: 4D MUSIC CMR: value-based imaging of neonates and infants with congenital heart disease
Source: J Cardiovasc Magn Reson. 2017 Apr 3;19:40. doi: 10.1186/s12968-017-0352-8 (PMC5376692; doi:10.1186/s12968-017-0352-8)
Supplement: Supplementary file 1 — Table S1. Image quality scoring criteria. Table S2. Primary diagnoses of neonates and infants undergoing MUSIC CMR. Table S3. Hemodynamic Variation Before and After Injection of Ferumoxytol. (DOCX 47 kb) [file 12968_2017_352_MOESM1_ESM.docx]

**4D MUSIC CMR: Value-Based Imaging of Neonates and Infants with Congenital Heart Disease**

| **Table S1. Image quality scoring criteria** | |
| --- | --- |
| **Structure** | **Score criteria*** |
| Atria  Ventricle | 1 Not evaluable due to gross motion artifact and borders not defined  2 Non-uniform blood pool signal and wall motion artifact precludes confident evaluation of luminal contents  3 Uniform blood pool signal with mild wall motion artifact adequate for confident visualization of luminal contents  4 Uniform blood pool signal with no motion artifact such that the ventricular walls, septum, papillary muscles and trabeculae are sharply defined |
| Interatrial and interventricular septum | 1 Not visualized  2 Presence of septal tissue is seen  3 Probable septal continuity but small defects cannot be confidently excluded  4 Definite septal continuity and small defects can be confidently excluded |
| Tricuspid apparatus  Mitral apparatus | 1 Not visualized due to gross motion artifact  2 Annulus visualized but borders poorly defined and cannot be confidently measured  3 Annulus clearly visualized and can be confidently measured but leaflets blurred  4 Annulus sharply defined and can be confidently measured and leaflets clearly visualized |
| LVOT, AV, and aortic root  RVOT, PV | 1 Not evaluable due to gross motion artifact and borders not defined  2 Outflow tract and annulus visualized but borders poorly defined and cannot be confidently measured  3 Outflow tract and annulus sharply defined and can be confidently measured but leaflets blurred  4 Outflow tract and annulus sharply defined and can be confidently measured and leaflets clearly visualized |
| Ascending aorta  Pulmonary artery (MPA to second order branch PA) | 1 Not evaluable due to gross motion artifact with non-uniform luminal signal  2 Uniform luminal signal with poor definition of the wall due to motion  3 Uniform luminal signal with mild blurring of the wall due to motion  4 Uniform luminal signal with no motion blurring and sharply defined wall |
| Coronaries | 1 Not evaluable due to gross motion artifact with visualization  2 Only origin of RCA and left main coronary can be identified  3 Origin and proximal course of RCA and LAD can be confidently visualized  4 Origin, proximal, and mid-course of the RCA and LAD and proximal takeoff of LCx can be confidently visualized |
| *AV, aortic valve; LAD, left anterior descending artery; LCx, left circumflex artery; LVOT, left ventricular outflow tract; MPA, main pulmonary artery; PA, pulmonary artery; PV, pulmonic valve; RCA, right coronary artery; RVOT, right ventricular outflow tract*  **Scores of 1 or 2 are considered non-diagnostic whereas scores of 3 or 4 are considered diagnostic* | |

| **Table S2. Primary diagnoses of neonates and infants undergoing MUSIC CMR** | | |
| --- | --- | --- |
| **Primary Diagnoses*** | **Neonates**  **(n=20)** | **Infants**  **(n=20)** |
| Anomalous coronary artery | 1 | 0 |
| Aortic coarctation or arch hypoplasia | 2 | 1 |
| Coronary aneurysms or arteritis | 0 | 2 |
| DORV | 1 | 3 |
| D-TGA with double aortic arch | 1 | 0 |
| Ebstein’s anomaly s/p BTS | 0 | 1 |
| Hemitruncus arteriosus | 0 | 1 |
| Heterotaxy /interrupted IVC | 0 | 1 |
| Heterotaxy /SV | 1 | 0 |
| Hypoplastic left heart syndrome | 2 | 0 |
| IAA /VSD | 3 | 0 |
| Intracardiac mass | 1 | 0 |
| PV dysplasia | 1 | 0 |
| Shone syndrome s/p Norwood-Stansel and bilateral Glenn | 0 | 1 |
| RV aneurysm | 0 | 1 |
| TAPVR or PAPVR | 2 | 2 |
| TOF-PA | 6 | 2 |
| TOF-PS | 1 | 1 |
| Vascular ring | 3 | 6 |
| **Each patient may qualify for more than one category.*  *D-TGA, dextro transposition of the great arteries; IAA, interrupted aortic arch; IVC, inferior vena cava; PA, pulmonary atresia; PAPVR, partial anomalous pulmonary venous return; PS, pulmonic stenosis; PV, pulmonic valve; SV, single ventricle; TAPVR, total anomalous pulmonary venous return; TOF, Tetralogy of Fallot; VSD, ventricular septal defect* | | |

| **Table S3. Hemodynamic Variation Before and After Injection of Ferumoxytol** | | | | |
| --- | --- | --- | --- | --- |
|  | **Immediately pre-injection** | **Immediately post-injection** | **30 minutes post-injection** | **>1 hour post-injection** |
| Heart rate (bpm) | 130 (124-134) | 128 (124-134) | 123 (120-138) | 130 (123-141) |
| SBP (mmHg) | 53 (50-55) | 53 (50-60) | 50 (50-55) | 55 (51-55) |
| DBP (mmHg) | 28 (25-30) | 28 (25-30) | 30 (25-31) | 30 (30-30) |
| Pulse oximetry (%) | 96 (94-98) | 96 (94-98) | 95 (92-98) | 98 (97-98) |
| End-tidal CO_2_ (mmHg) | 31 (28-36) | 33 (30-37) | 34 (32-35) | 35 (30-38) |
| ***bpm*** *beat per minute;* ***DBP*** *diastolic blood pressure;* ***SBP*** *blood pressure*  *Values are reported as median and 25-75^th^ interquartile. P values for all parameters were >0.05 using Analysis of Variance for repeated measures.* | | | | |
