# Supplementary material for: 4D MUSIC CMR: value-based imaging of neonates and infants with congenital heart disease
Source: J Cardiovasc Magn Reson. 2017 Apr 3;19:40. doi: 10.1186/s12968-017-0352-8 (PMC5376692; doi:10.1186/s12968-017-0352-8)

**4D MUSIC CMR: Value-Based Imaging of Neonates and Infants with Congenital Heart Disease**

**Figure S1.** Multiplanar volume rendered MUSIC images from a 12-month old infant (11.4 kg) boy with a double aortic arch and complete vascular ring are shown in A-C. Breath-held first pass FE-CMRA (A) shows ill-defined right atrial border (scored 2) and blurring of the left ventricular (LV) endocardial border as well as the LV outflow tract (scored 1). Although breath-held steady state FE-CMRA (B) shows more complete filling of the cardiac chambers, the borders remain blurred. Steady state 4D MUSIC (C) shows clear take-off of the proximal left main coronary artery (green arrow), sharp intra-cardiac borders (scored 4), clear definition of the aortic valve leaflets (red arrow), and left pulmonary artery (white arrow). Additional multiplanar volume rendered MUSIC images from a 3-day old neonate (2.6 kg) boy with Tetralogy of Fallot and pulmonary atresia are shown in D-E. Both coronary ostia are well visualized (orange and red arrows). The course of the proximal to mid right coronary artery (RCA) is well defined. The proximal left main coronary artery (LCA, orange arrow [D] and red arrow [E]) arises from the left coronary cusp. A large perimembranous ventricular septal defect is present (green arrow) along with an overriding aorta. Ao, aorta; LV, left ventricle; RV, right ventricle


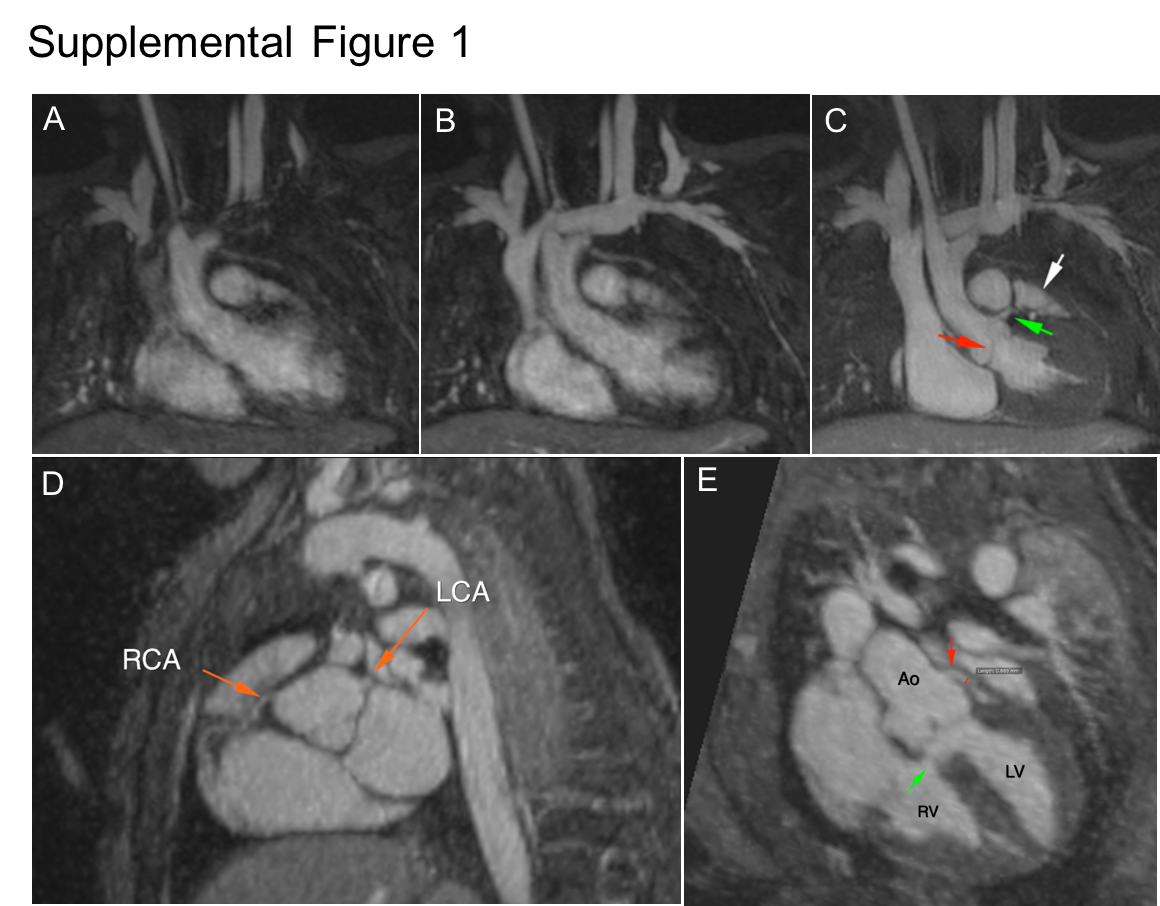

Supplement: Supplementary file 2 — Figure S1. Multiplanar volume rendered MUSIC images from a 12-month old infant (11.4 kg) boy with a double aortic arch and complete vascular ring are shown in A-C. (DOCX 793 kb) [file 12968_2017_352_MOESM2_ESM.docx]
